# Apple SERRATE negatively mediates drought resistance by regulating MdMYB88 and MdMYB124 and microRNA biogenesis

**DOI:** 10.1038/s41438-020-0320-6

**Published:** 2020-07-01

**Authors:** Xuewei Li, Pengxiang Chen, Yinpeng Xie, Yan Yan, Liping Wang, Huan Dang, Jing Zhang, Lingfei Xu, Fengwang Ma, Qingmei Guan

**Affiliations:** State Key Laboratory of Crop Stress Biology for Arid Areas/Shaanxi Key Laboratory of Apple, College of Horticulture, Northwest A&F University, Yangling, 712100 Shaanxi PR China

**Keywords:** Drought, Plant molecular biology

## Abstract

The function of serrate (SE) in miRNA biogenesis in *Arabidopsis* is well elucidated, whereas its role in plant drought resistance is largely unknown. In this study, we report that *MdSE* acts as a negative regulator of apple (*Malus* × *domestica*) drought resistance by regulating the expression levels of *MdMYB88* and *MdMYB124* and miRNAs, including mdm-miR156, mdm-miR166, mdm-miR172, mdm-miR319, and mdm-miR399. MdSE interacts with MdMYB88 and MdMYB124, two positive regulators of apple drought resistance. MdSE decreases the transcript and protein levels of MdMYB88 and MdMYB124, which directly regulate the expression of *MdNCED3*, a key enzyme in abscisic acid (ABA) biosynthesis. Furthermore, MdSE is enriched in the same region of the *MdNECD3* promoter where MdMYB88/MdMYB124 binds. Consistently, *MdSE* RNAi transgenic plants are more sensitive to ABA-induced stomatal closure, whereas *MdSE OE* plants are less sensitive. In addition, under drought stress, MdSE is responsible for the biogenesis of mdm-miR399, a negative regulator of drought resistance, and negatively regulates miRNAs, including mdm-miR156, mdm-miR166, mdm-miR172, and mdm-miR319, which are positive regulators of drought resistance. Taken together, by revealing the negative role of MdSE, our results broaden our understanding of the apple drought response and provide a candidate gene for apple drought improvement through molecular breeding.

## Introduction

Serrate (SE) in *Arabidopsis* is a conserved eukaryotic RNA processing factor that was first reported to mediate the formation of early juvenile leaves and phase length^1^. Encoding a C_2_H_2_ zinc-finger protein, SE is required for normal shoot development^2^. Moreover, SE influences the alternative splicing of pre-mRNAs that primarily affect the selection of alternative 5′ splice sites of first introns^3^. Other genes with alternative splicing affected by SE encode transcription factors, splicing factors, and stress-related proteins^3^. SE also functions in intron splicing and the transcription of intronless genes by pausing and elongating polymerase II complexes to promote their association with these intronless target genes^4,5^. Moreover, label-free quantitative proteomic analysis has revealed that SE is regulated by abscisic acid (ABA) under flooding stress^6^. In addition to these functions, SE has a role in microRNA (miRNA) biogenesis^7,8^, and previous studies report that SE and Hyponastic Leaves1 (HYL1) form a complex with DICER1 to achieve efficient and precise processing of pri-miRNAs^9^.

Drought stress is a major limiting factor that affects the yield and quality of apple. Researchers have long sought to increase the drought resistance of apple trees using molecular tools, such as genetic transformation and QTL mapping of loci associated with water use efficiency^10–14^. To date, a number of genes have been reported to play positive or negative roles in apple drought resistance. For example, MdMYB88 and MdMYB124 are proven to be two positive regulators of apple drought stress that influence xylem formation and secondary cell wall deposition^11^. In addition, MdMYB88 and MdMYB124 bind to gene promoters containing the *cis*-element AACCG^11,13^ to regulate expression.

miRNAs are ~20–25 nucleotide (nt) endogenous small molecules involved in various plant processes, including development and environmental stresses^15–17^. For example, the overexpression of miR156 improves the drought tolerance of alfalfa (*Medicago sativa*) by silencing *SPL13*^18^, a squamosa promoter-binding-like protein that binds to a core GTAC sequence in the promoter region of dihydroflavonol-4-reductase (*DFR*) to induce anthocyanin biosynthesis in response to drought stress^18,19^. miR393 is involved in the rice response to stress by targeting auxin receptors and plays negative roles in drought and salt stress^20^. In addition, transgenic *Arabidopsis* plants overexpressing miR399 exhibit hypersensitivity to drought but enhanced tolerance to salt stress and exogenously applied ABA^21^. In the apple genome, 23 conserved, 10 less conserved, and 42 apple-specific miRNAs or families with distinct expression patterns have been identified; these miRNAs target various genes and represent a wide range of enzymatic and regulatory activities^22^. Genome-wide miRNA analysis has revealed that 61 and 35 miRNAs are differentially expressed in drought-tolerant and drought-sensitive apple hybrid progeny, respectively, under drought stress^23^. Among these mdm-miRNAs, mdm-miR156 and mdm-miRn249 are two positive regulators of apple osmotic stress^23^.

ABA is a drought-induced phytohormone that plays important roles in plant responses to environmental stresses. Upon drought stress, ABA accumulates rapidly to promote stomatal closure and avoid water loss^24,25^. Exogenous ABA treatment effectively and sufficiently upregulates many stress-marker proteins in wheat and maize that are indicated to enhance drought tolerance^26,27^. ABA also acts as a signaling molecule in response to drought stress. Rice (*Oryza sativa*) OsPM1 (PLASMA MEMBRANE PROTEIN1) encodes an ABA influx carrier that mediates the movement of ABA across the plasma membrane and plays important roles in drought responses^28^. Under drought conditions, elevated ABA induces the production of H_2_O_2_ in guard cells, and subsequent H_2_O_2_-activated Ca^2+^ channels mediate the influx of Ca^2+^ in intact guard cells to close stomata^29,30^.

In the current study, we provide evidence that MdSE participates in the drought resistance of apple by negatively regulating MdMYB88- and MdMYB124-mediated ABA homeostasis. MdSE also regulates the expression of miRNAs that play critical roles in drought resistance in apple. Our results highlight the roles of MdSE in the drought tolerance of apple and thereby provide genetic determinants for apple breeding.

## Results

### MdSE interacts with and reduces the transcript and protein levels of MdMYB88 and MdMYB124

**Fig. 1:**
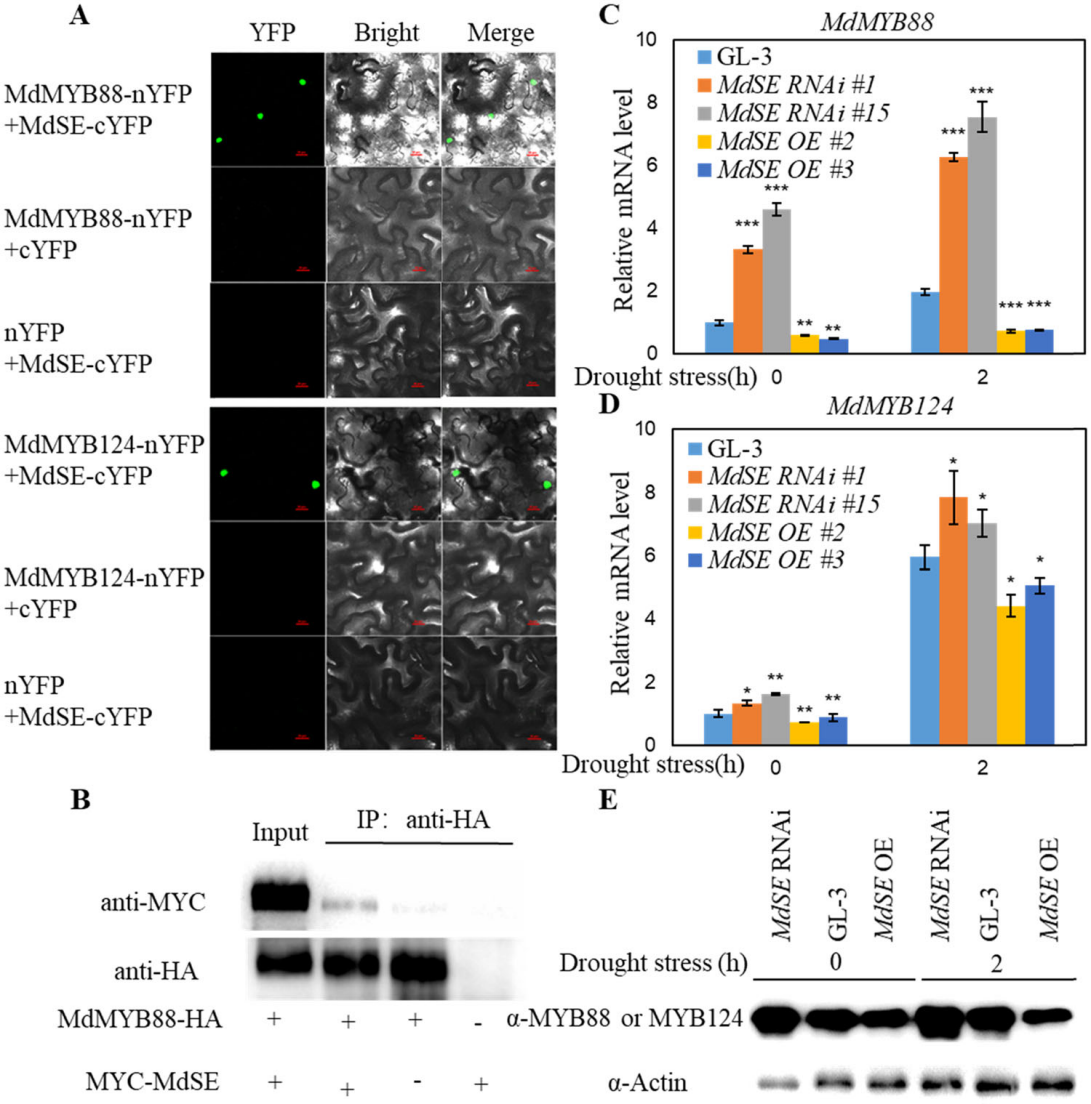
MdSE reduces the transcript and protein levels of MdMYB88 and MdMYB124 by interacting with them. **a** MdSE interacts with MdMYB88 and MdMYB124 in tobacco leaves by BiFC analysis. Bars = 20μm. **b** MdSE interacts with MdMYB88 in tobacco by Co-IP analysis. MdMYB88-HA was coinfiltrated with MYC-MdSE in tobacco leaves. Proteins were extracted and immunoprecipitated with an anti-HA antibody. The immunocomplex was then detected with western blot using anti-MYC or anti-HA. **c**, **d** Expression level of *MdMYB88* or *MdMYB124* in GL-3, *MdSE* RNAi, and *MdSE* OE plants under control or drought conditions. Data are means ± SD (*n* = 3). Student’s *t* test was performed, and statistically significant differences are indicated by *P < 0.05, **P < 0.01, or ***P < 0.001. **e** Protein level of MdMYB88 or MdMYB124 in GL-3, *MdSE* RNAi, and *MdSE* OE plants under control or drought conditions

When applying affinity-purified mass spectrometry analysis to ascertain the interacting partners of MdMYB88 and MdMYB124, the SERRATE protein, which usually participates in miRNA biogenesis, pri-miRNA, and pre-mRNA splicing in *Arabidopsis*, was identified^3^. The interaction between MdSE and MdMYB88 or MdMYB124 was confirmed by BiFC analysis (Fig. 1a), which was further verified by a Co-IP assay (Fig. 1b). However, MdSE did not interact with MdMYB88 in yeast, as demonstrated by a yeast two-hybrid analysis (Fig. S1), indicating that the physical interaction of MdSE with MdMYB88 or MdMYB124 may require another component.

The expression of *MdSE* was examined in MdMYB88 and MdMYB124 transgenic plants, which were generated previously^13^. qRT-PCR analysis revealed no regulation of *MdSE* by MdMYB88 or MdMYB124 under control or dehydration conditions (Fig. S2). To assess whether *MdMYB88* or *MdMYB124* expression levels are regulated by MdSE, *MdSE* RNAi and *MdSE* OE plants were generated. The transgenic plants were verified at the DNA and RNA levels (Fig. S3). After air dehydration for 2 h, transcripts of *MdMYB88* or *MdMYB124* were reduced dramatically in *MdSE* OE plants but increased in *MdSE* RNAi plants (Fig. 1c, d). Western blot analysis confirmed the downregulation of MdMYB88 and MdMYB124 by MdSE under drought (Fig. 1e), indicating that under drought conditions, MdSE decreases levels of MdMYB88 and MdMYB124 proteins.

Since SE is responsible for the alternative splicing of pre-mRNAs in *Arabidopsis*, we then examined the transcripts of *MdMYB88* and *MdMYB124* in *MdSE* RNAi plants by a RT-PCR assay. We found that decreased *MdSE* levels did not affect splicing of *MdMYB88* and *MdMYB124* in apple under control or drought conditions (Fig. S4).

### MdSE subcellular localization and expression pattern

**Fig. 2:**
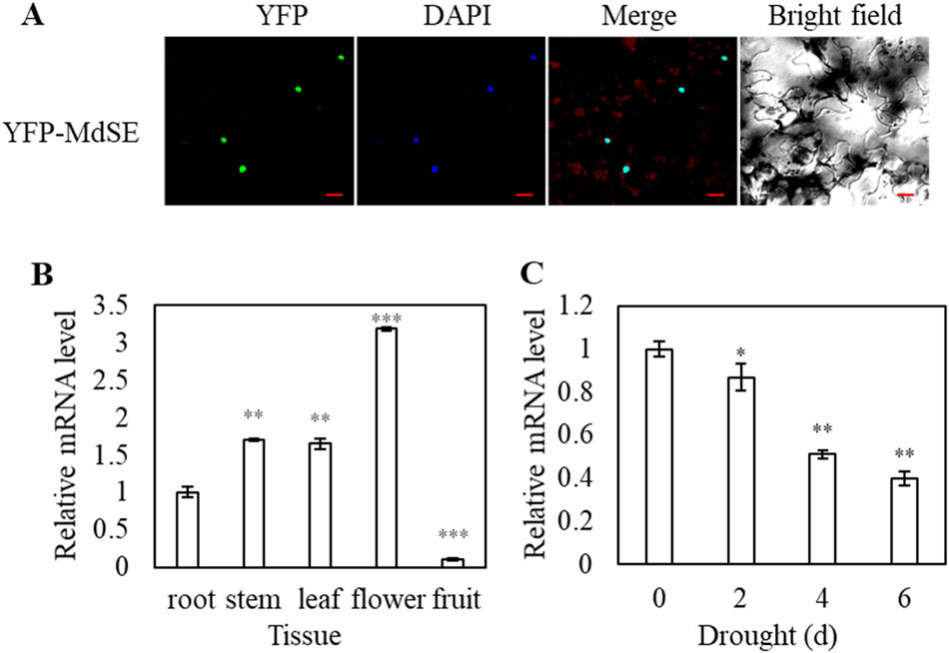
MdSE localization and expression patterns. **a** MdSE is localized in the nucleus. Bars = 20μm. **b** Expression of *MdSE* in different organs in *M. prunifolia*. **c** Transcript level of *MdSE* in response to drought. Error bars indicate the standard deviation (*n* = 3 in **b** and **c**). Student’s *t* test was performed, and statistically significant differences are indicated by *P < 0.05, **P < 0.01, or ***P < 0.001

Protein alignment demonstrated that MdSE shares 67.2% sequence similarity with *Arabidopsis* SE and is more closely related to SERRATE from *Prunus persica* (Fig. S5). Based on a transient expression assay, the YFP–MdSE fusion protein was present in the nucleus of tobacco cells (Fig. 2a), consistent with the nuclear localization of SE in *Arabidopsis*^31^. SE from *M. prunifolia* was found to be expressed predominantly in flowers, followed by stems, leaves, and roots (Fig. 2b). The *MdSE* expression level was reduced in response to drought stress (Fig. 2c).

### MdSE is a negative regulator of drought tolerance

**Fig. 3:**
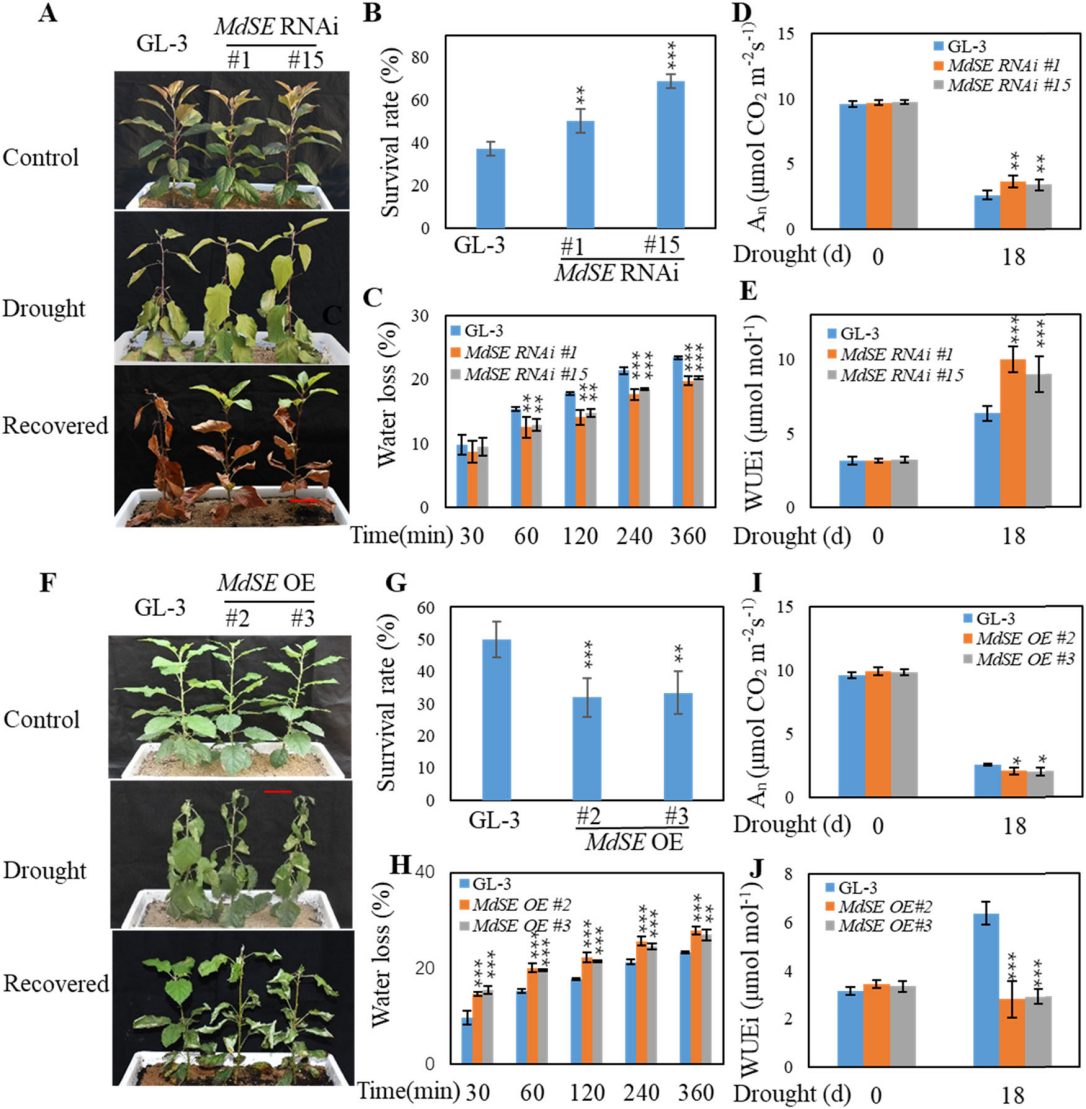
MdSE transgenic plant response to drought stress. **a**, **f** Drought tolerance of GL-3 and transgenic plants. Bars = 5cm. Five-month-old *MdSE* OE and GL-3 plants were treated with drought for 24 days, and then rewatered for 7 days. *MdSE* RNAi and GL-3 plants were treated with drought for 30 days, and then rewatered for 7 days. **b**, **g** Survival rate of GL-3 and transgenic plants shown in **a** and **f**. Data are means ± SD (*n* = 36). **c**, **h** Water loss of GL-3 and *MdSE* OE or RNAi plants under dehydration conditions for up to 360 min. Data are means ± SD (*n* = 10). **d**, **i** The rate of photosynthesis (A_N_). **e**, **j** Intrinsic water use efficiency (WUEi). Data are means ± SD (*n* = 15). Student’s *t* test was performed, and statistically significant differences are indicated by *P < 0.05, **P < 0.01, or ***P < 0.001

To understand the biological function of MdSE in the drought response of apple, the drought tolerance of 5-month-old *MdSE* transgenic plants and nontransgenic GL-3 plants was examined. After withholding water for 30 days, and then rewatering for 7 days, 50–70% of *MdSE* RNAi plants survived, whereas only 40% of the control plants were alive (Fig. 3a, b). Water loss from *MdSE* RNAi plants was much lower than loss from GL-3 plants (Fig. 3c). Compared with GL-3 plants under drought for 18 days, *MdSE* RNAi plants had higher photosynthesis rates and water use efficiency (Fig. 3d, e).

When exposed to drought for 24 days and rewatered for 7 days, ~30% of 5-month-old *MdSE* OE plants survived; in contrast, 50% of GL-3 plants remained alive (Fig. 3f, g), which suggested that *MdSE* OE plants are more sensitive to drought stress. In addition, *MdSE* OE plants lost significantly more water under dehydration (Fig. 3h), and *MdSE* OE plants had significantly lower photosynthesis rates and water use efficiency than GL-3 plants under drought (Fig. 3i, j). Together, these data suggest that *MdSE* negatively regulates apple drought resistance.

### MdSE regulates stomatal aperture and ABA accumulation under drought

**Fig. 4:**
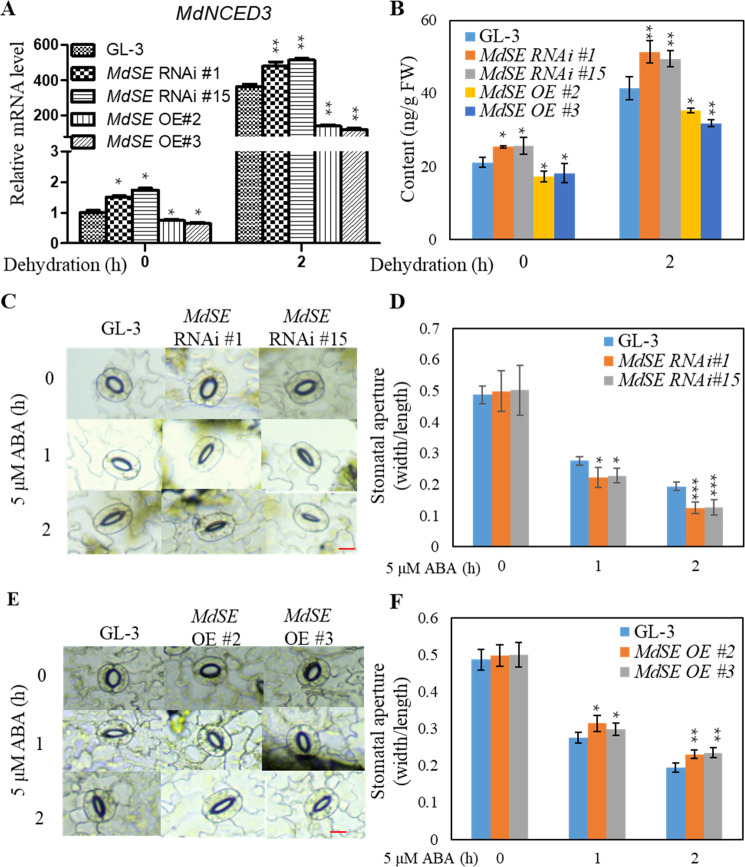
ABA response and content in MdSE RNAi and MdSE OE plants. **a** Expression of *MdNCED3* in GL-3, *MdSE* RNAi, or *MdSE* OE plants under control or dehydration conditions. Error bars indicate standard deviation (*n* = 3). Student’s *t* test was performed, and statistically significant differences are indicated by *P < 0.05 or **P < 0.01. **b** The ABA content in GL-3, *MdSE* RNAi, or *MdSE* OE plants under control or dehydration conditions. Error bars indicate standard deviation (*n* = 5). **c**, **e** Representative images of stomata of GL-3 and *MdSE* transgenic plants in response to ABA treatment. Bars = 10 μm. **d**, **f** Stomatal aperture of GL-3 and *MdSE* transgenic plants under ABA treatment. Data are the means ± SD; 5 leaves were used, and at least 80 stomatal apertures were measured for each treatment. Student’s *t* test was performed, and statistically significant differences are indicated by *P < 0.05, **P < 0.01, or ***P < 0.001

Because MdSE interacts with MdMYB88 and MdMYB124 in vivo (Fig. 1) and MdMYB88 and MdMYB124 directly regulate 9-cis-epoxycarotenoid dioxygenase 3 (*MdNCED3*) expression and ABA content under drought conditions (unpublished), MdSE regulation of *MdNCED3* transcripts and ABA content under drought was investigated. According to qRT-PCR analysis, drought-induced *MdNCED3* expression was significantly higher in *MdSE* RNAi plants but lower in *MdSE* OE plants than in GL-3 plants (Fig. 4a). The ABA content was then measured in *MdSE* transgenic and GL-3 plants under control and drought conditions. LC-MS analysis showed that *MdSE* RNAi plants contained significantly more ABA but that *MdSE* OE plants contained less ABA in response to drought than GL-3 plants (Fig. 4b). Consistently, *MdSE* RNAi plants were hypersensitive to ABA-induced stomatal closure, whereas *MdSE* OE plants were less sensitive (Fig. 4c–f).

### MdSE is enriched in the *MdNCED3* promoter

**Fig. 5:**
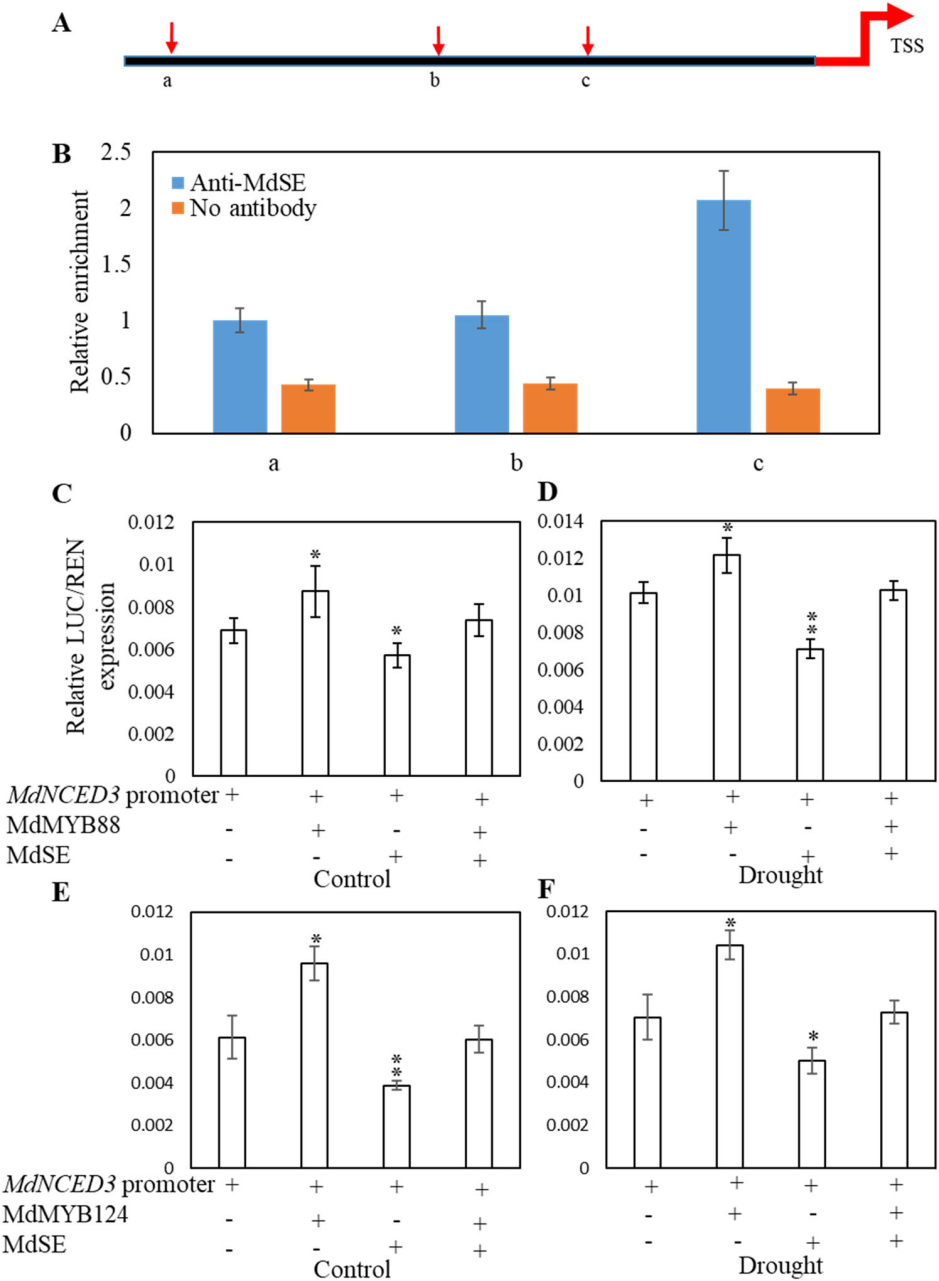
MdSE is enriched in the MdNCED3 promoter and decreases MdNCED3 activity. **a** Diagram of *MdNCED3* promoter regions. **a**–**c** Represent fragments containing two *cis*-elements of AGCCG from −1830 to −1826 bp, −1368 to −1364 bp and one *cis*-element of CGCGG from −880 to −876 bp, respectively. For the negative control, no antibody was added. TSS transcription start site. **b** MdSE enrichment in the MdNCED3 promoter determined by ChIP-qPCR analysis. **c**–**f** Relative luciferase activity from dual luciferase reporter assays in *N. benthamiana* leaves. Pro35S::REN was used as an internal control. Quantification was performed by normalizing firefly luciferase activity to that of Renilla luciferase. Leaves of *N. benthamiana* were coinfiltrated and grown for 72 h, and then leaves were collected or dehydrated for an additional 2 h. Error bars indicate standard deviation (*n* = 10). Student’s *t* test was performed, and statistically significant differences are indicated by *P < 0.05 or **P < 0.01

Regulation of the main biosynthetic pathway of ABA is mediated by NCED3, which cleaves 9-cis-epoxy-carotenoids and produces in the active isomer of ABA^32^. Because the ABA content of *MdSE* transgenic lines was affected under drought conditions, we investigated whether *MdSE* influences the *MdNECD3* expression level by associating with the *MdNCED3* promoter. Chromatin immunoprecipitation (ChIP) experiments were carried out using an SE-specific antibody, followed by qRT-PCR with the same primers used to assess MdMYB88 and MdMYB124 binding activity to *MdNCED3*. The ChIP-qPCR results showed enrichment of MdSE at the *MdNCED3* promoter in the same region where MdMYB88 and MdMYB124 bind (Fig. 5a, b). A dual luciferase reporter assay was also used to detect the influence of MdSE on *MdNCED3* expression. The results showed that MdMYB88 and MdMYB124 enhanced *MdNCED3* expression under both normal and dehydration conditions, whereas MdSE reduced the expression of *MdNCED3*. When MdSE was present, the expression of *MdNCED3* induced by MdMYB88 or MdMYB124 was attenuated under control and drought conditions (Fig. 5c–f). These results suggest that the regulation of *MdNCED3* by MdSE depends on its association with MdMYB88 and MdMYB124.

### MdSE regulates the biogenesis of miRNAs in apple under drought

**Fig. 6:**
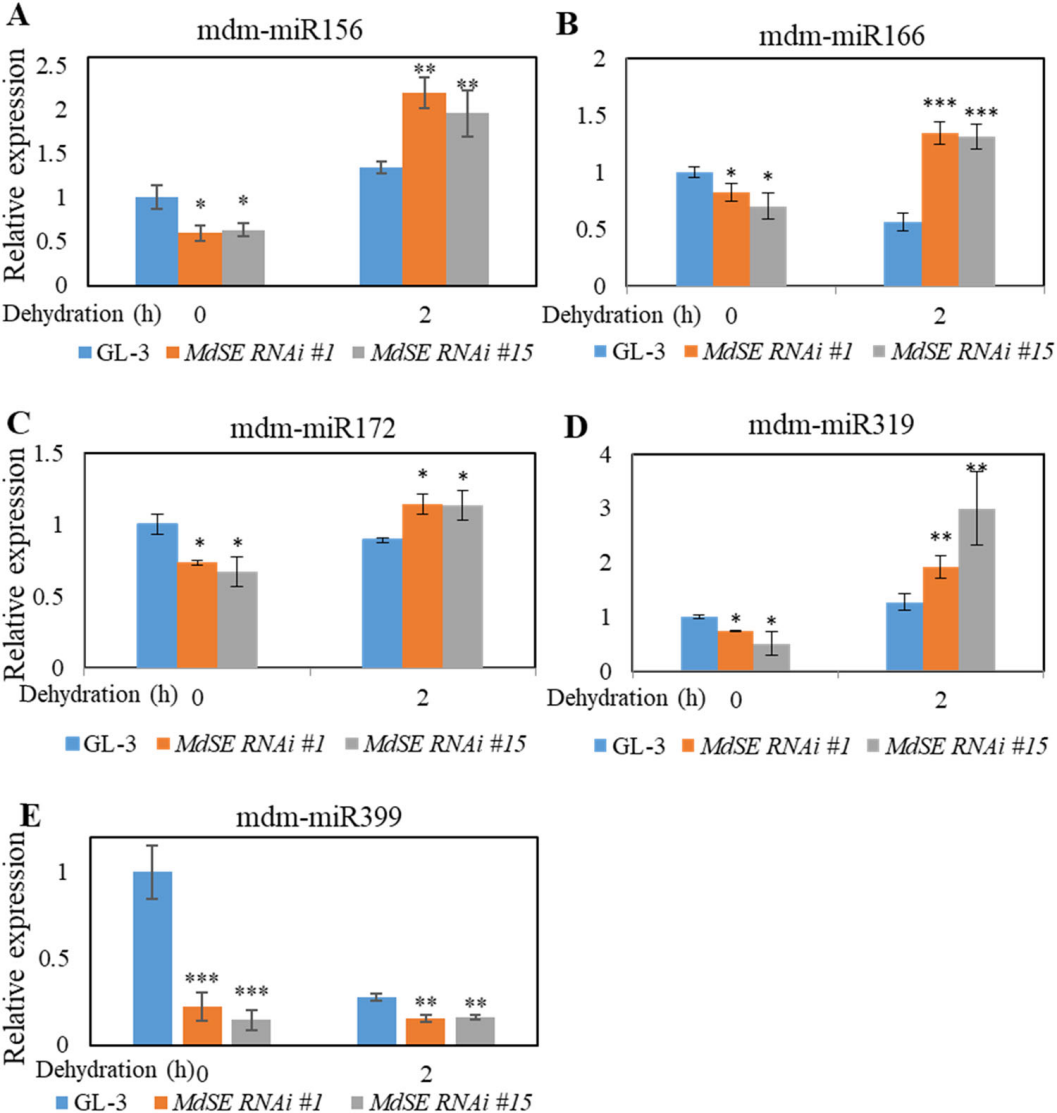
MdSE affects the biogenesis of drought-responsive miRNAs in apple in response to drought. Mdm-miR156 (**a**), mdm-miR166 (**b**), mdm-miR172 (**c**), mdm-miR319 (**d**) and mdm-miR399 (**e**) were determined by stem-loop qPCR. Error bars indicate standard deviation (*n* = 3). Student’s *t* test was performed, and statistically significant differences are indicated by *P < 0.05, **P < 0.01, or ***P < 0.001

*Arabidopsis* SE is required for the biogenesis of miRNA^33,34^. Loss-of-function mutant plants of SE have upward curling and radialized leaves^35^; secondary inflorescences lack an associated cauline leaf, and inflorescences often produce siliques that emerge from the same node on the stem^34^. Curly leaves on *MdSE* RNAi plants were not observed (Fig. 3), which may be due to ~30–50% MdSE being maintained in *MdSE* RNAi plants (Fig. S3A). To understand whether *MdSE* has a similar function to *Arabidopsis* SE in miRNA biogenesis, we analyzed the expression of drought-responsive miRNAs, including mdm-miR156, mdm-miR166, mdm-miR172, mdm-miR319, and mdm-miR399, using stem-loop qPCR. Mdm-miR156, miR166, miR172, and miR319 are positive regulators of apple osmotic stress^36^ and drought resistance in alfalfa^18^, *Arabidopsis*^37,38^, and creeping bentgrass^39^, though miR399 is a negative regulator of drought resistance^21^. The expression levels of these miRNAs were reduced in *MdSE* RNAi plants under control conditions (Fig. 6), suggesting a similar role for MdSE and SE in miRNA biogenesis. Under drought stress, compared with GL-3 plants, the expression levels of mdm-miR156, mdm-miR166, mdm-miR172, and mdm-miR319 increased in *MdSE* RNAi plants, whereas mdm-miR399 expression was reduced (Fig. 6), consistent with the drought tolerance phenotype of *MdSE* RNAi plants. These data indicate that *MdSE* has a similar function in miRNA biogenesis as *Arabidopsis* SE and negatively modulates drought through regulation of drought-responsible miRNAs.

## Discussion

In this study, we characterized a protein interacting with MdMYB88 and MdMYB124, MdSE, in response to drought. MdSE plays a negative role in apple drought resistance by negatively modulating the expression of MdMYB88 and MdMYB124, leading to the downregulation of *MdNCED3* and reduced ABA levels. Furthermore, MdSE regulates the expression of drought-responsive miRNAs under drought stress, which may contribute to its negative role in drought resistance.

The interaction between MdSE and MdMYB88 or MdMYB124 was confirmed by BiFC and Co-IP analyses. However, an in vitro interaction was not verified by Y2H analysis, indicating the possibility of a bridge between MdSE and MdMYB88. For example, the interaction of *Arabidopsis* C-terminal domain phosphatase-like 1 (CPL1) and HYL1 requires SE as a bridge^31^. MdHYL1 is a putative candidate for bridging MdSE and MdMYB88 or MdMYB124. Indeed, we found that MdHYL1 interacts with MdSE in Y2H analysis (Fig. S6). The interaction between SE and HYL1 was also observed in *Arabidopsis*^33^, indicating that the functions of SE and HYL1 in some plant processes are conserved among plant species. Factors other than MdHYL1 might act as a bridge between MdSE and MdMYB88 or MdMYB124, but further study is necessary.

SE is responsible for alternative splicing of pre-mRNAs^3^. In addition to the regulation of *MdMYB88* and *MdMYB124* transcripts by MdSE (Fig. 1), we hypothesized that MdSE might modulate the alternative splicing of *MdMYB88* and *MdMYB124*. However, RT-PCR results showed that reduced *MdSE* levels did not affect splicing of *MdMYB88* and *MdMYB124* in apple under control and drought conditions (Fig. S4). It is possible that MdSE can affect alternative splicing of other genes under control or drought conditions. The only challenge of alternative splicing detection in apple is that 30–50% of MdSE was still functional in *MdSE* RNAi plants (Fig. S3A), which might affect the accuracy of analyses.

We demonstrated that MdSE is a negative regulator of drought resistance. First, survival ability analysis suggested that *MdSE* RNAi plants were more tolerant to drought stress but that *MdSE* OE plants were more sensitive (Fig. 3). Second, under dehydration conditions, *MdSE* OE plants lost water more quickly than *MdSE* RNAi plants (Fig. 3c, h). Third, under drought stress, the cell membranes of *MdSE* RNAi plants were less damaged, as indicated by ion leakage (Fig. 3). Fourth, MdSE negatively regulated ABA accumulation and some drought-positive miRNAs (Figs. 4 and 6a–d). In addition, MdSE positively regulated drought-negative mdm-miR399. Fifth, *Arabidopsisse-1* mutants were also more tolerant to drought stress (Fig. S7). All these data support that MdSE plays a negative role in apple drought resistance and that the role of SE under drought might be conserved among plant species.

ABA is a plant stress hormone that regulates stomatal closure within and outside guard cells through combinational mechanisms^40^. In *Arabidopsis*, maize, wheat, rice, and apple, elevated ABA content contribute to plant drought tolerance by inducing stomatal closure and stress-related signal transduction^21,23,24,26,27,41,42^. NCED3 is considered to be the key contributor to ABA production under water deficit conditions, and ZEP and AAO3 play minor roles^43,44^. In *Arabidopsis*, short vegetative phase (SVP) is able to bind to the promoters of the ABA catabolism pathway genes *CYP707A1*, *CYP707A3*, and *AtBG1*, and thus contributes to ABA homeostasis^45^. Homeostasis of ABA was also regulated by reversible glycosylation mediated by ABA-UGTs (uridine diphosphate glucosyltransferases) to affect ABA bioactivity (Fig. S8)^46,47^. In our study, more ABA accumulated in *MdSE* RNAi transgenic lines compared with *MdSE* OE and GL-3 plants (Fig. 4b). Such negative regulation of ABA content should contribute to the negative role of MdSE in drought resistance. We also found that MdSE was enriched at the promoter region of *MdNCED3*, which is the same region bound by MdMYB88 and MdMYB124 (Fig. 5). Considering the in vivo interaction between MdSE and MdMYB88 or MdMYB124, we conclude that the enrichment of MdSE at the *MdNCED3* promoter was due to recruitment by MdMYB88 or MdMYB124 instead of direct binding.

*Arabidopsis* SE is a critical component required for pri-miRNA processing and miRNA biogenesis^33,34^. The results of microarray analysis showed that numerous miRNAs and their target genes are misexpressed in *se-1*, including miR156, miR165, miR167, miR163, miR164, miR168, and miR171^5,33^. In our study, stem-loop qPCR analysis demonstrated reduced levels of mdm-miR156, mdm-miR166, mdm-miR172, mdm-miR319, mdm-miR399, and mdm-miR398 transcripts in *MdSE* RNAi transgenic lines under normal environmental conditions (Fig. 6), indicating a conserved role for SE in miRNA biogenesis among plant species.

miRNAs participate in various plant processes, including root development^48^, flowering time^49^, apical dominance^35^, and plant architecture^16^, and are also associated with tolerance to environmental stresses, including salt^50^, drought^18,21^, cold^51^, and bacterial infection^36^. The overexpression of mdm-miR156 in apple calli enhances osmotic stress^22^ and drought stress tolerance in alfalfa (*Medicago sativa*)^18^; miR166, miR172, and miR319 are also reported to act as positive regulators of drought tolerance in rice^38^, soybean^52^, and creeping bentgrass (*Agrostis stolonifera*)^39^, and miR399 plays a negative role in *Arabidopsis* drought resistance^21^. In our study, mdm-miR156, mdm-miR166, mdm-miR172, and mdm-miR319 were induced in *MdSE* RNAi transgenic plants after drought exposure, whereas mdm-miR399 was reduced, suggesting that these factors might contribute to the drought tolerance of *MdSE* RNAi plants.

In summary, our study elucidated the roles of *MdSE* in drought stress resistance. MdSE plays a negative role in drought resistance by affecting miRNA biogenesis and negatively regulating protein accumulation of MdMYB88 and MdMYB124, which results in negative regulation of ABA accumulation. Our study provides a deeper understanding of the complex mechanism of MdSE in response to drought stress and identifies a candidate gene for drought improvement through molecular breeding.

## Materials and methods

### Plant materials and growth conditions

For gene cloning, “Golden delicious” (*Malus* × *domestica*) grown in a greenhouse was used for RNA extraction. GL-3, a genotype selected from seedlings of “Royal Gala” (*Malus* × *domestica*), was used for genetic transformation^53^. GL-3 grown on Murashige and Skoog (MS) medium (4.43 g/L MS salts, 30 g/L sucrose, and 7 g/L agar, pH 5.8) supplemented with 0.2 mg/L 6-benzylaminopurine and 0.2 mg/L indoleacetic acid (IAA) under long-day conditions (14 h light/10 h dark cycle) for 4 weeks at 25 °C were used for gene transformation. *MdMYB88/124* RNAi plants and *MdMYB88* or *MdMYB124* overexpression plants were produced in a previous study^13^. The transgenic plants were rooted in MS medium (2.22 g/L MS salts, 20 g/L sucrose, 7.5 g/L agar, 0.5 mg/L IAA, 0.5 mg/L indolebutyric acid (IBA), pH 5.8) for 2 months, and then transplanted to substrate (Pindstrup, Denmark). *se-1* was obtained from ABRC.

### RNA extraction and qRT-PCR analysis

Detailed methods for RNA extraction and qRT-PCR analysis are provided in ref. ^13^. The primers used for qRT-PCR analysis are listed in Supplementary Table 1.

### Generation of transgenic apple

To generate a construct for *MdSE* overexpression, the coding region (CDS) of *MdSE* was cloned into pGWB414 to produce MdSE-pGWB414. To knock down *MdSE*, a 292-bp fragment of *MdSE* was introduced into pK7WIWG2D, resulting in *MdSE*-pK7WIWG2D. Both plasmids were transformed into *Agrobacterium* strain EHA105. For genetic transformation, we used an *Agrobacterium*-mediated transformation method. Plant transformation was carried out according to Dai et al.^53^. Briefly, 4-week-old GL-3 leaves were cut into strips in liquid MS medium (4.43 g/L MS salts and 30 g/L sucrose, pH 5.2) with EHA105 (OD_600_ = 0.6–0.9) carrying the relevant plasmid for 15 min. Then, the leaf strips were transferred into maintenance medium (4.43 g/L MS + 2 mg/L TDZ + 0.5 mg/L NAA + 100 μM acetosyringone +1 mM betaine +7.5 g/L agar +30 mg/L sugar, pH = 5.8). After 3 days, leaf strips were transferred into selection medium (4.43 g/L MS + 2 mg/L TDZ + 0.5 mg/L NAA + 250 mg/L cefotaxime +50 mg/L kanamycin +7.5 g/L agar +30 mg/L sugar, pH = 5.8) for 4 weeks in the dark, and then incubated for 6 weeks under light. The transgenic buds that stayed green on selection medium were grown for ~4 weeks. DNA and RNA were extracted from the transgenic plants and GL-3 and used to detect transgene insertion and *MdSE* expression levels by PCR and RT-qPCR, respectively. Transgenic plants with transgene insertion, as well as altered expression levels of *MdSE*, were selected for further experiments. The primers used are listed in Supplementary Table 1.

### Drought treatment

Drought treatment was carried out by withholding water for a certain number of days, and then rewatering for 7 days, followed by calculation of the survival rate. Specifically, 5-month-old *MdSE* OE and GL-3 plants were treated with drought for 24 days, and then rewatered for 7 days. *MdSE* RNAi and GL-3 plants were treated with drought for 30 days, and then rewatered for 7 days. To obtain photosynthesis data (the rate of photosynthesis and intrinsic water use efficiency), a LiCor-6400 portable photosynthesis system (LiCor) was used.

Detached leaves from 5-month-old *MdSE* OE, *MdSE* RNAi, and GL-3 were used for the water loss assay.

### Stomatal aperture measurements

For stomatal aperture measurements, we used leaves of 2-month-old soil-grown transgenic apple and GL-3 plants. Leaves were cut off and plunged into stomatal opening solution (30 mM KCl, 0.1 mM CaCl_2_, and 10 mM MES-KOH, pH 6.15) under light (120 μmol m^−2^ s^−1^) for 2 h to induce stomatal opening as described^54^. Then, ABA was added to the stomatal opening solution to a final concentration of 5 μM. The leaf epidermis was observed for stomatal aperture with an EX30 microscope (SDPTOP) after ABA treatment for 1 or 2 h. Stomatal length and width were measured by ImageJ software, and stomatal aperture was then calculated.

### Western blot

Proteins were extracted from leaf samples with extraction buffer (50 mM Tris-HCl, pH 7.5, 150 mM NaCl, 2 mM EDTA, 10% glycerol, 1% NP-40, 1 mM phenylmethylsulfonyl fluoride). Twenty micrograms of protein was separated by 10% SDS–PAGE and blotted onto PVDF membranes (Millipore) using standard methods. The blots were blocked for 2 h in PBS (50 mM Na_2_HPO_4_, pH 7.4) with 5% nonfat milk, after which anti-MdMYB88 and MdMYB124 or anti-actin (ABclonal, AC009) antibodies were added. After 2 h, the blots were washed twice in PBS milk, and a secondary antibody (goat anti-rabbit horseradish peroxidase-conjugated, 1 mg/mL; catalog no. HS101; Transgen Biotech) was added. After washing, the blots were treated with Bio-Rad ChemiDoc XRS+ to visualize the signals.

### Yeast two-hybrid assay

A yeast two-hybrid assay was carried out according to the manufacturer’s manuals (Clontech, 630439, 630489). MdMYB88–155 aa and CDS of *MdSE* were introduced into pGBDT7. CDS of MdMYB88 or MdHYL1 was cloned into pGADT7. MdSE-pGBDT7 and MdMYB88-pGADT7 or MdHYL1-pGADT7 were cotransformed into yeast strain AH109. MdMYB88–155 aa-pGBDT7 and MdHYL1-pGADT7 were also cotransformed into yeast strain AH109. Positive clones were selected on SD/-Leu-Trp, and then on SD/-Leu-Trp-His-Ade + x-α-gal plates for the x-α-gal assay. The primers used are listed in Supplementary Table 1.

### Subcellular localization, BiFC, and Co-IP assays

To generate constructs for BiFC assays, we cloned the CDS of *MdMYB88* and its paralog gene *MdMYB124* into pSPYNE-35S; the CDS of *MdSE* was cloned into the pSPYCE-35S vector. For subcellular localization, the CDS of *MdSE* was cloned into the pEearleyGate104 vector. Transient expression assays were performed according to Xie et al.^13^. After 3 days, fluorescent signals in transformed tobacco leaves were then detected using a Nikon A1R/A1 confocal microscope (Nikon).

For Co-IP analysis, the CDS of *MdMYB88* was amplified by PCR and cloned into pEarleyGate 101; the CDS of *MdSE* was cloned into pEarleyGate 203. Co-IP analyses were performed as described previously^55^.

The primers used are listed in Supplementary Table 1.

### ChIP-qPCR

ChIP-qPCR assays were performed as described previously^13^. Tissue-cultured GL-3 was used for crosslinking, and the ChIP assay was performed with an anti-SE antibody (Agrisera, AS09 532A). Three regions of the *MdNCED3* promoter were examined by qPCR, with no antibody ChIP samples serving as the reference. The primers used for ChIP-qPCR are listed in Supplementary Table 1.

### ABA measurement

ABA was extracted as described^56^. Frozen apple leaf samples (about 100 mg fresh weight) was ground in liquid nitrogen, and then extracted with 1 ml of cold extraction buffer (methanol:isopropanol:acetic acid = 20:79:1, v/v/v). After centrifugation at 4 °C and 12,000 rpm for 10 min, the supernatant was transferred into a 2 mL tube, and 500 μL cold extraction buffer was added followed by vortexing for 5 min. The extraction process was repeated three times followed by centrifugation at 4 °C and 12,000 rpm for 10 min. The supernatant was filtered through a 0.22 μm PTFE filter (Waters, Milford, MA, USA). GC-MS analysis was carried out using a QTRAP^®^ 5500 LC-MS/MS (AB SCIEX, Redwood City, USA).

## Accession numbers

Sequence data can be found in NCBI under the following numbers: MdMYB88 (KY569647), MdMYB124 (KY569648), MdSE (KY568649), and MdNCED3 (XM_008380174.2).

## Supplementary information

Supplemental Figures

Supplemental Table 1
