# Supplementary material for: Apple SERRATE negatively mediates drought resistance by regulating MdMYB88 and MdMYB124 and microRNA biogenesis
Source: Hortic Res. 2020 Jul 1;7:98. doi: 10.1038/s41438-020-0320-6 (PMC7326988; doi:10.1038/s41438-020-0320-6)
Supplement: Supplementary file 1 — Supplemental Figures [file 41438_2020_320_MOESM1_ESM.pdf]

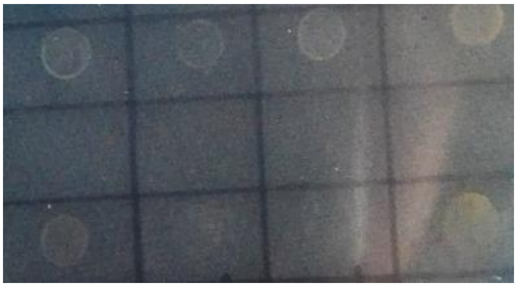

Figure S1 MdSE does not interact with in yeast. The CDS of *MdSE* and *MdMYB88* were introduced into pGBDT7 and pGADT7, respectively. Positive clones were selected on SD medium (-Leu-Trp-His-Ade + x-α-gal) to identify protein interactions.

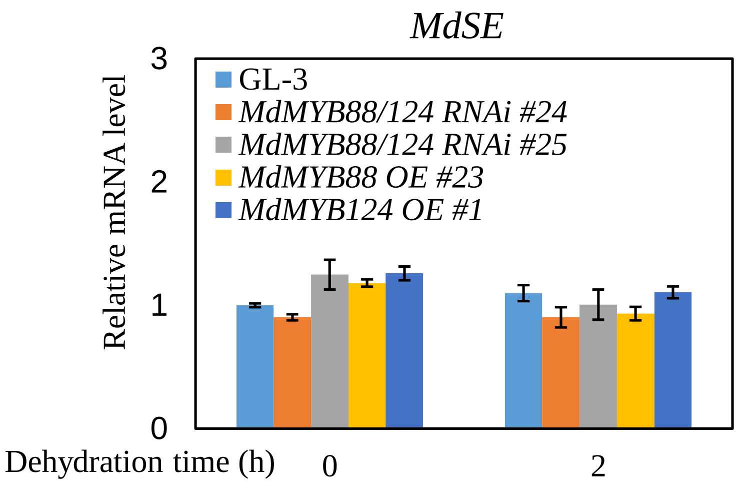

Figure S2 MdMYB88 and MdMYB124 do not regulate *MdSE* transcript under control or drought conditions. Transcript level of *MdSE* in *MdMYB88* OE, *MdMYB124* OE and *MdMYB88/124* RNAi plants. Data are means ± SD (n = 3). Student's *t* test was performed and statistically significant differences were indicated by * ($P<0.05$), ** ($P<0.01$) or *** ($P<0.001$).

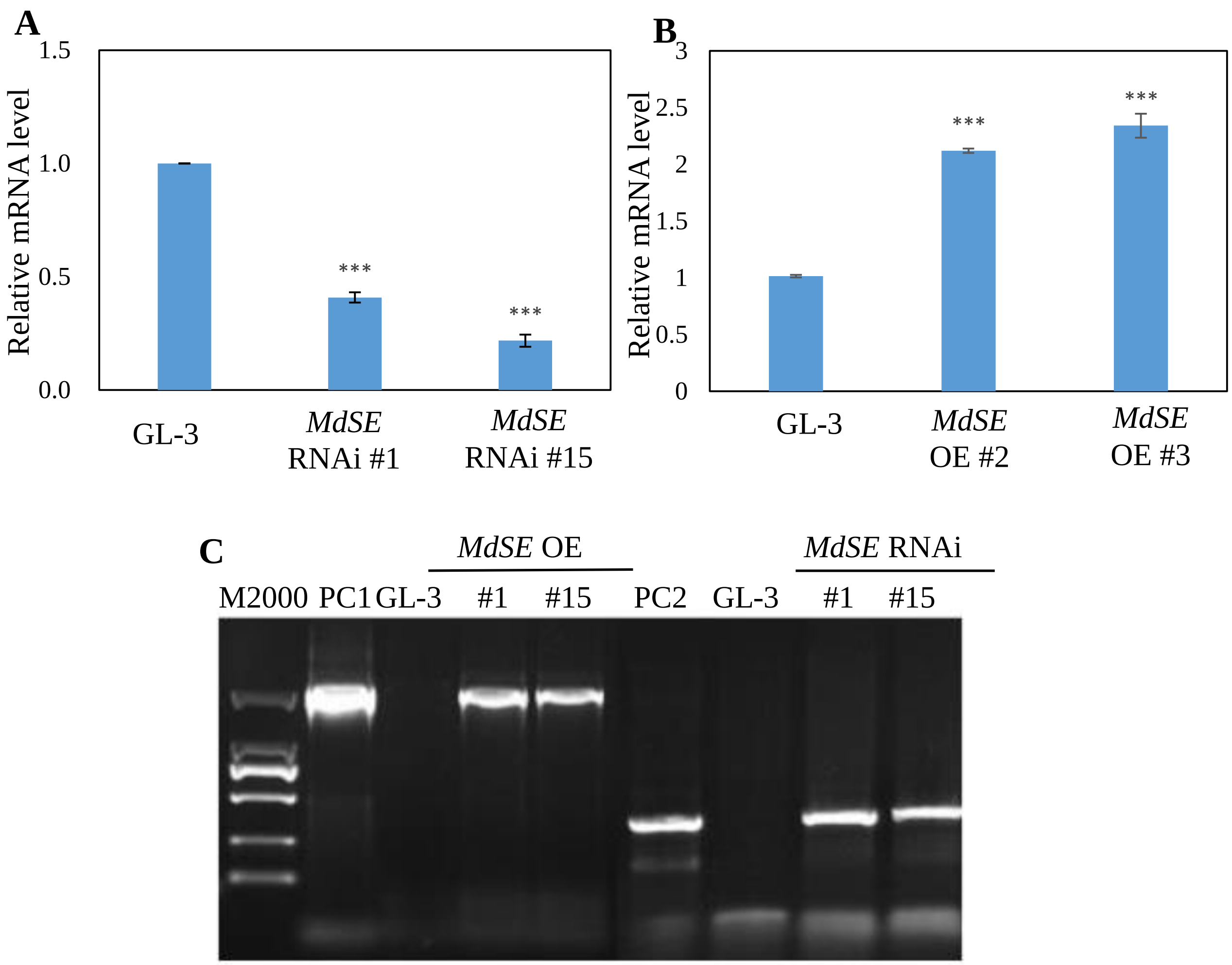

Figure S3 Identification of *MdSE* RNAi or OE transgenic plants. (A) and (B) Transcript level of *MdSE* in *MdSE* RNAi (A) and OE (B) plants. Data are means ± SD (n = 3). Student's *t* test was performed and statistically significant differences were indicated by * (P<0.05), ** (P<0.01) or *** (P<0.001). (C) Detection of *MdSE* RNAi or OE transgenic plants at DNA level. M2000, DNA Marker 2000. PC1, positive control with MdSE-pGWB414 as a template. PC2, positive control with MdSE-pK7GWIWG2D as a template.

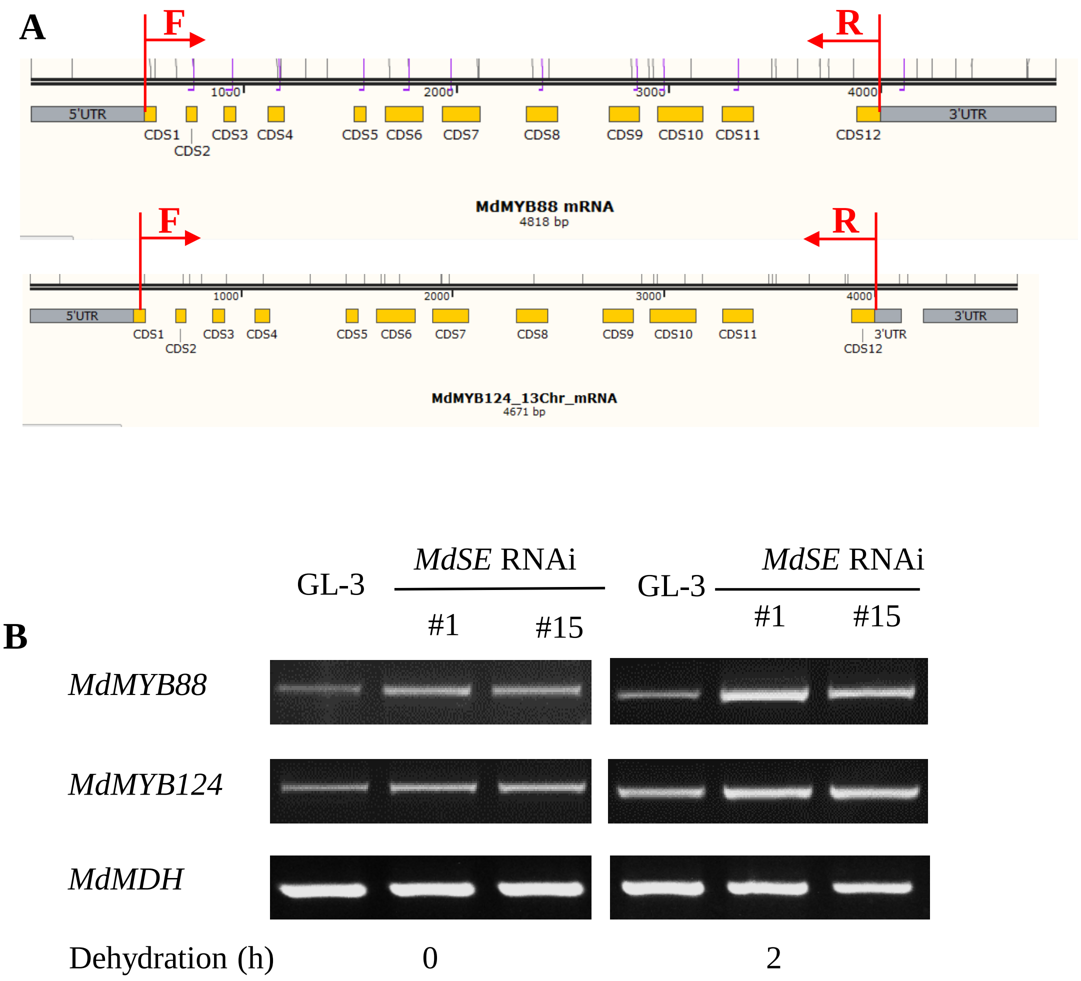

Figure S4 MdSE does not affect splicing of *MdMYB88* or *MdMYB124*. (A) The gene structure of *MdMYB88* and *MdMYB124*. The yellow squares represent exons, the blanks represent introns, and the grey squares were untranslated regions. The red lines marked the primers (forward primer and reverse primer) used for RT-PCR in (B). (B) *MdMYB88* or *MdMYB124* transcript in *MdSE* RNAi plants under control and drought conditions. Total RNA was extracted and reversely transcribed into the first strand cDNA which was then amplified using primers in (A). MdMDH was used as a control.

A

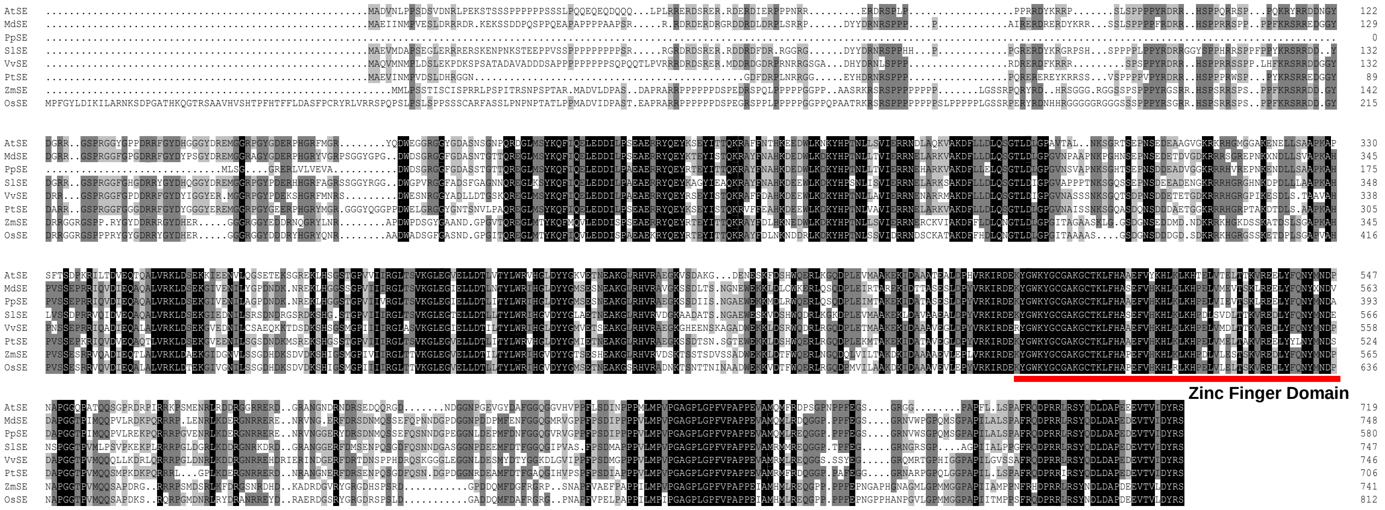

B

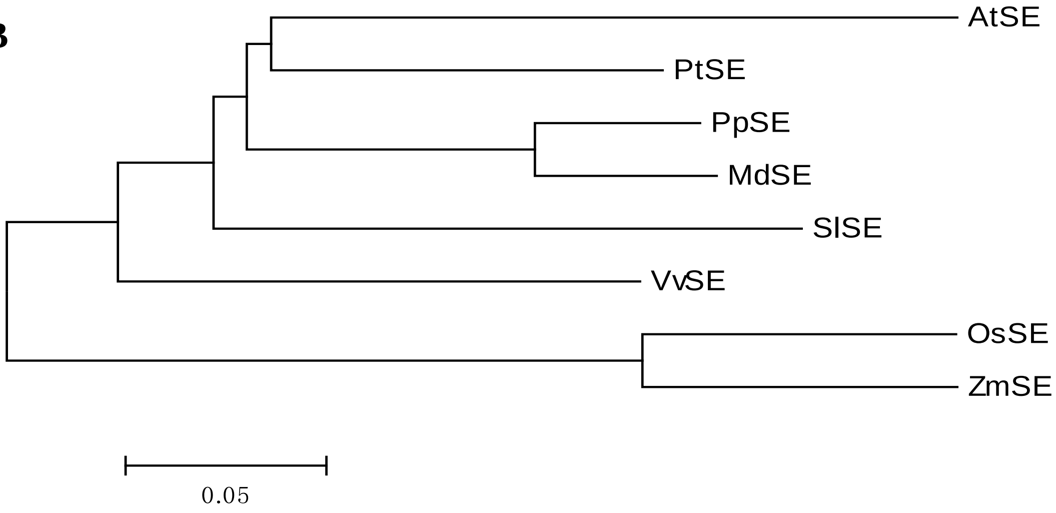

Figure S5 Sequence similarity and phylogenetic tree of SE from different plant species. (A) Multiple sequence alignment of SE in different plant species, including *Arabidopsis thaliana* (At), *Malus domestica* (Md), *Populus trichocarpa* (Pt), *Solanum lycopersicum* (Sl), *Vitis vinifera* (Vv), *Fragaria vesca* (Fv), *Oryza sativa* (Os), and *maize* (Zm). The underlined amino acids represent zinc-finger domain. (B) Phylogenetic tree of SE from different plant species.

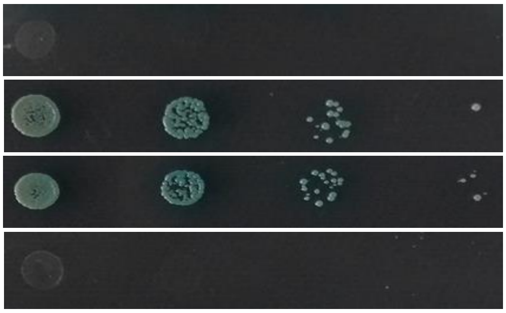

Figure S6 MdHYL1 interacts with MdMYB88 and MdSE. MdMYB88-155aa which lacks self-activation and full length MdSE were introduced into pGBDT7, respectively. The CDS of MdHYL1 and MdMYB88 were introduced into pGADT7. Positive clones were selected on SD meida (-Leu-Trp-His-Ade + x-α-gal) to identify protein interactions.

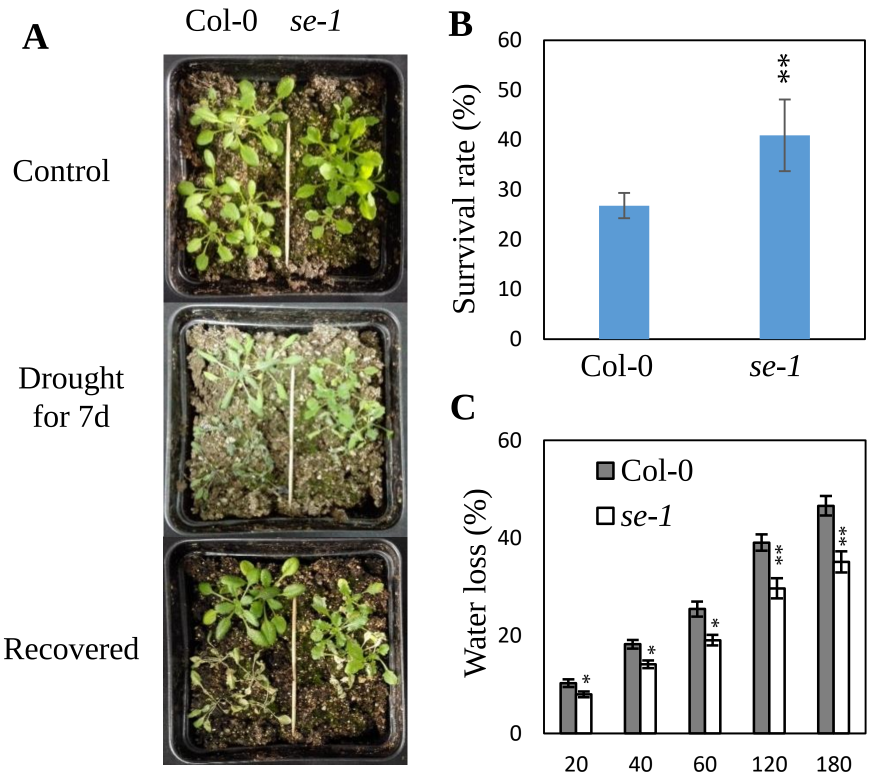

Figure S7. Phenotype of *se-1* mutants under drought stress. (A) Phenotypes of *se-1* mutants under drought. Water was withheld for 12 d, and rewaterd for 7 d to calculate survival rate. (B) Survival rate of *se-1* mutants under drought stress. Data are means ± SD (n = 20). (C) Water loss rate of *se-1* mutants under dehydration conditions. Data are means ± SD (n = 10). Student's *t* test was performed and statistically significant differences were indicated by * (P<0.05) or ** (P<0.01).

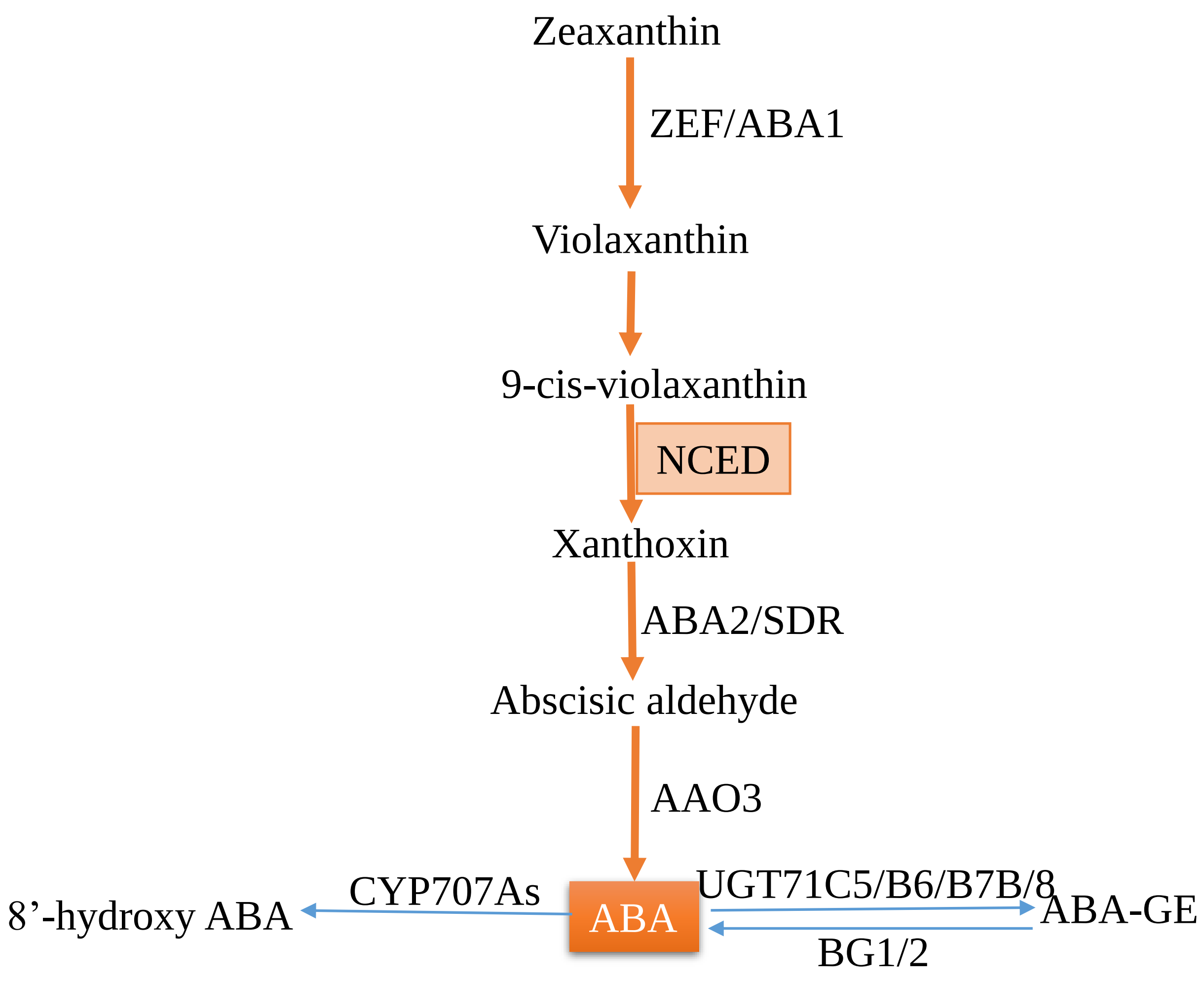

Figure S8. A simplified pathway for biosynthesis of abscisic acid (ABA). The *de novo* biosynthesis process of ABA is catalyzed by a series of enzymes including ZEF/ABA1 (zeaxanthin epoxidase), NCED (9-cis-epoxycarotenoid dioxygenases), ABA2/SDR (ABA-deficient 2) and AAO3 (ABA-aldehyde oxidase 3). The degradation process of ABA is catalyzed mainly by two ways, one is catalyzed by CYP707As (cytochrome P450 707A subfamily monooxygenases) to 8’-hydroxy ABA, and the other one is catalyzed by UGT71C5/B6/B7/B8 (UDP-glucosyltransferase) to ABA-GE which can also be converted to ABA by BG1/2 (glycosyl hydrolase).
